# Taenia saginata, the incidental find in case of intestinal perforation after blunt trauma and literature review

**DOI:** 10.1016/j.ijscr.2023.107909

**Published:** 2023-01-27

**Authors:** Behzad Nematihonar, Seyed Pedram Kouchak Hosseini, Alireza Haghbin Toutounchi

**Affiliations:** Department of General Surgery, Imam Hosein Medical and Educational Center, Shahid Beheshti University of medical sciences, Tehran, Iran

**Keywords:** Case report, Hollow viscus, Taeniasis, Intestinal perforation, Blunt trauma

## Abstract

**Introduction and importance:**

*Taenia saginata* (*T. saginata*) is one of the most common cestodes in humans. Isolated perforation of the small bowel after blunt abdominal trauma is infrequent and the diagnosis should be based on exclusion.

**Case presentation:**

We report a case of a 34-year-old man who presented following a car-pedestrian accident. Clinical examinations and symptoms suggested an acute abdomen. Investigations led to the diagnosis of hollow viscus perforation, so emergency laparotomy was performed. At the exploration of peritoneal cavity, unexpectedly, a live tapeworm was found in the peritoneal cavity. The perforation was repaired and medication were continued by anthelmintic. He was discharged with good condition.

**Clinical discussion:**

We discuss this rare incidental finding in a patient with bowel perforation and suggest the taeniasis as a possible cause of intestinal perforation. The medical literature and reviews have been searched to find more information about taeniasis and its cause-effect in GI tract complications.

**Conclusion:**

Increasing the public knowledge about food hygiene and encouraging eat well-cooked meat can control the cycle of transmission of cestodes. Taeniasis should be considered a possible cause of intestinal obstruction or perforation, especially in endemic areas.

## Highlights

• Taenia saginata, the incidental find in the case of abdominal blunt trauma with Intestinal Perforation.
• Isolated perforation of the small bowel after blunt abdominal trauma.
• Taeniasis should be considered as a cause of acute abdomen.
• The intestinal perforation has remained a rare complication in parasitic diseases.

## 1 Introduction

*Taenia saginata* (*T. saginata*) is one of the humans' most pathogenic cestodes [1]. This tapeworm can infect humans with undercooked meat such as beef, pork, or fish [2]. Infected individuals can remain asymptomatic for years, and the symptoms may be only the impulsive passage of proglottids.

[3] According to reviews, Taeniasis remains a problem in developing countries and our region MENA (middle east and north Africa) [4], [14]. Intestinal perforation has remained a rare complication in parasitic diseases [5].

*Imam* Hosein medical and educational center is the biggest referral center for trauma in the east of Tehran, Iran. This case is reported by the department of general surgery of this hospital.

This article is modified according to the SCARE 2020 criteria [16].

## 2 Presentation of case

### 2.1 History

A 34-year-old man was presented as a car-pedestrian accident to emergency department. Based on ATLS in trauma assessment, primary survey was done. Vital signs were BP: 110/80, PR: 90 Saturation of oxygen: 96. He was not completely conscious (GCS: 8). We Resuscitated and intubated him. He had not active bleeding or ecchymosis. His father (source of information) did not remember any relevant past medical or surgical history. His drug history was adverse. They had no familial history of specific chronic diseases or any cancer. He was not married and lived with his parents. They were low in the socioeconomic state. He was educated and had no psychological history.

### 2.2 Assessment

Because of his loss of consciousness (GCS = 8), the abdominal examination was not assessable. FAST ultrasound was done, and it was negative for free fluid in the abdomen and pelvis. His vital signs were stable. His temp was 37 °C. His relevant first lab results were WBC: 18/Hb: 15. For other aspects of injury, CT scan of brain and thorax was done.

His brain CT scan showed diffuse axonal brain injury. CT scan of the thorax was cleared, and there was no pneumothorax or rib fracture. In the second FAST ultrasound after 30 min checking, mild inter-loop fluid was seen. Whether the next plan was CT scan with IV contrast enhancement, but because of country problems and some medical sanctions on IRAN, we did not have the contrast agent, therefor diagnostic peritoneal lavage (DPL) was performed. Food particles were found in DPL fluid. So for urgent laparatomy, he was taken to OR.

### 2.3 Operation summery

**Fig. 1:**
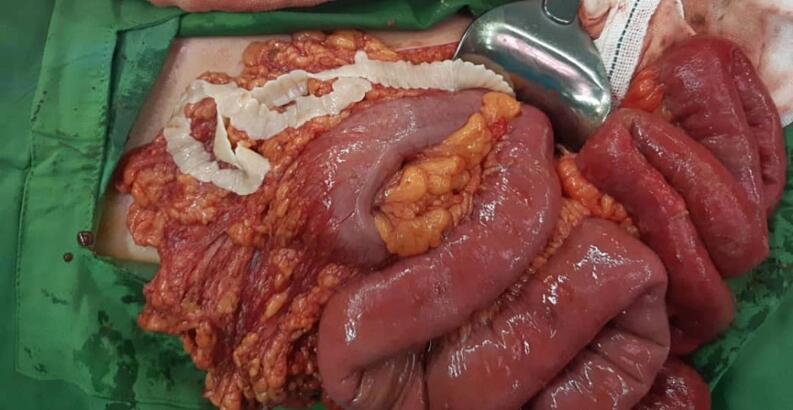
The cestode with length of 2 m in peritoneal cavity.

**Fig. 2:**
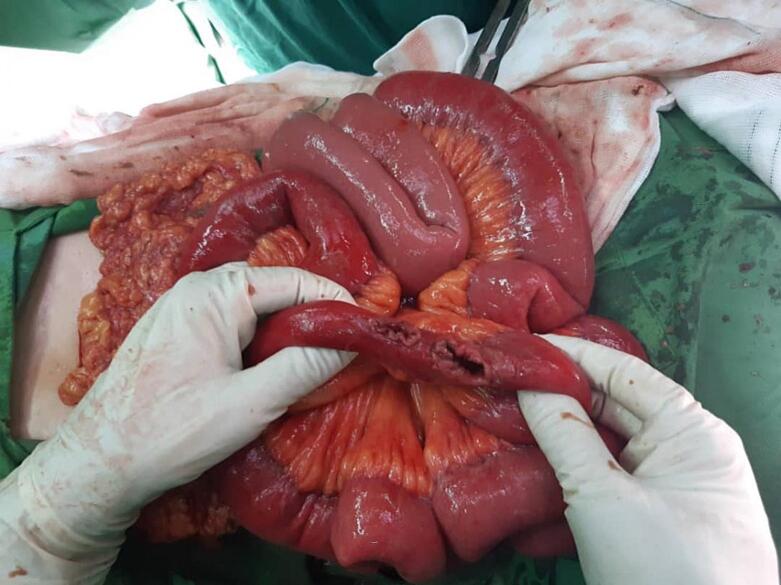
The bowel perforation site.

**Fig. 3:**
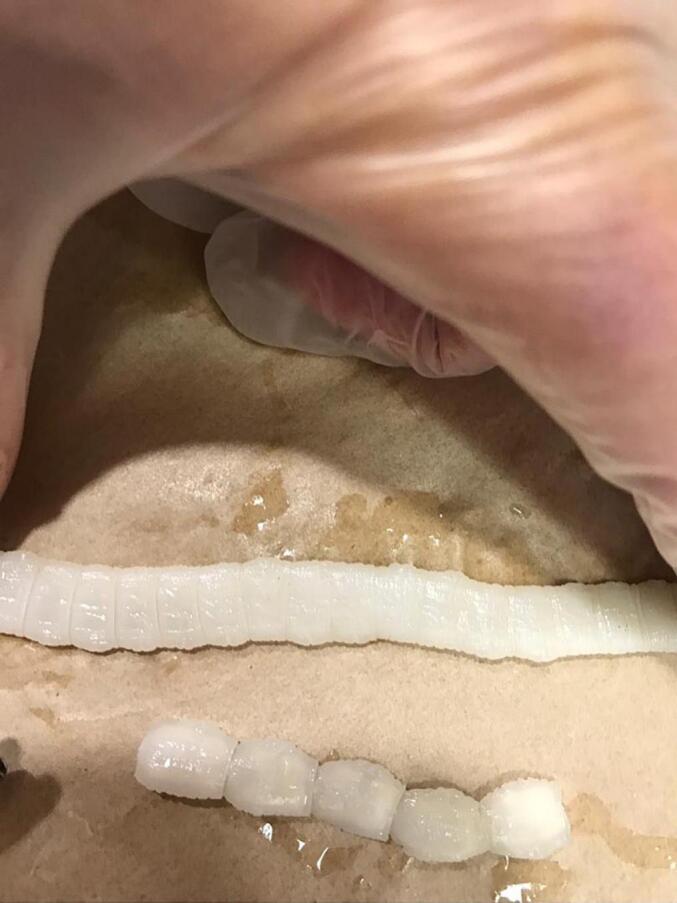
The cestodes.

Laparotomy under general anesthesia through mid-line incision was performed by an expert attending general surgeon and senior resident. In exploration of peritoneal cavity, 3 cm and 2 cm perforations were seen in 220 cm distal to the treitz ligament and 5 cm proximal to ileocecal valve, respectively. Unexpectedly, a 2-m length tape worm and also 2 cm and 5 cm worms were seen there. The peritoneal cavity was washed with warm normal saline, the first perforation site was resected and primary end-to-end anastomosis was performed. In addition, because of ileocecal valve damage, right hemicolectomy and side-to-side ileocolic anastomosis was also performed with linear cutter (LC) stapler. Finally, two hemovac drains were established close to anastomosis sites. Postoperatively, medical treatment with broad-spectrum intravenous antibiotics and anthelminthic therapy was started (Fig. 1, Fig. 2, Fig. 3).

### 2.4 Outcome

According to patient's state, he was admitted to ICU for three days and regained full consciousness. There was no sign of leakage or failing of the anastomoses. The intestinal function was normal, he tolerated regular diet with no nausea or vomiting, leukocytosis decreased, and he was discharged with well general condition and normal defecation function. After three weeks, the stool exam was clear and parasite eggs were not detected.

## 3 Discussion

*Taenia* spp. includes *T. saginata*, *T. solium* and *T. asiatica* which is very close in features to *T. saginata*. *T. saginata* is the most common tapeworm infestation in humans, which may cause gastrointestinal tract complications due to obstruction, perforation or anastomotic leakage [6]. Also, Ascariasis is known to be responsible for the higher incidence of intestinal complications than taeniasis. Ascariasis can cause small intestinal obstruction, biliary lesions, pancreatitis, appendicitis and peritonitis in children [12]. Cattle and buffalo are the intermediate hosts, and humans are the definitive host. It causes taeniasis in the human intestine, cysticercosis in cattle, and remains in the liver and muscles [7], [8]. Humans can become infected when they use bovine meat contaminated with cysticerci. Cysticerci matures into an adult worm in the human intestine that lives many years, about twenty in the host [7]. Morphology or a combination of microscopic, immunological and molecular methods provides high utility in differentiating Taenia species. Taenia can also be diagnosed by detecting serum antibodies against recombinant antigens, such as rTSES33 and rTSES38. Ether PCR is the gold standard for detecting and identifying different Taenia types [7], [10].

Taeniasis is usually asymptomatic; most of the time, patients complain of proglottids in the stool. However, sometimes mild constitutional symptoms and consolidation from infesting, like nausea, weight loss and abdominal pain and discomfort, happen. Infrequently, the tapeworm can lead to severe medical conditions such as jejunal perforation and peritonitis [6], [7]. So, it can be a severe surgical case.

The most surgical manifestation of taeniasis is abdominal pain, mall nutrition, obstruction, inflammation and perforation of the small intestine, appendix or colon [9]. Local inflammatory reactions or obstruction due to an impacted tapeworm, bowel perforation and consecutive peritonitis may occur [10]. Abdomen Blunt Trauma resulting in perforation or de-vascularization of the intestine has captured the intrigue of many surgeons through the years. The diagnosis of taeniasis is based on the patient's history and macroscopic&microscopic results of stool samples [10]. Early diagnosis and experimental treatment will be of utmost importance in managing these injuries.

Some articles reported taenia complications in humans, like perforation or obstruction, and one remarkable case reported gall bladder perforation by *T. saginata* [11]. The gastrointestinal tract wall is thickened and harder to be perforated easily by Taenia. Usually, there is a trigger that causes or prepares the wall for perforation.

Mechanical intestinal obstruction due to bolus of Taenia and mucosa contact, leading to gross inflammatory edema, is the most accepted theory for bowel perforation(the mechanical theory) [6]. This mechanism is favorable in the small intestine, especially in the ileocecal valve, where the luminal diameter is narrow and parasite–mucosa contact is more likely to happen [12]. According to this theory, less complication is expected in the large intestine. Also, Bhandari et al. Literature review makes this theory stronger. They have remarked that parasite-mucosa contact is the cause of local inflammation that predisposes to perforation. According to their analysis, the most reported perforation site is the small bowel Because the luminal diameter is narrowest there, as said above. In addition, they show that the incidence of perforation is higher in more prominent Taenia species (*saginata & asiatica*), and fewer happen with *T. solium*, but it causes systemic complications more [10].

Despite of usual nature of *T. saginata*, there is a theory about some treatment with medications or some peripheral trigger that can provoke and activate adult worms. So they perforate the tracked to invasion to other parts or scape from anthelmintic agents [13].

The standard treatment for all tapeworms is praziquantel, with a dose of 10 mg/kg orally in a single dose for any taeniasis [15].

Overall, there are no strong evidences in this case to show that there was a connection between intestinal rupture caused by trauma and Taeniasis.

DPL is a safe and cost-effective method of evaluating patients with potential intra-abdominal injury, but CT scan with IV and oral enhancement should be performed if available. In this case, we did not have the contrast agent because of limitations and sanctions. So DPL can be helpful in these situations.

## 4 Conclusion

Taeniasis and complications remain a health problem in developing countries. Increasing the public knowledge about food hygiene and encouraging to use of well-cooked meat can control the cycle of transmission of cestodes. Delay in diagnosing such infestations by cestodes is critical as it can lead to fatal outcomes. Cestodes in the bowel can cause obstruction, mall-nutrition, ischemia and sudden perforations. Taeniasis should be considered a possible cause of intestinal obstruction or perforation, especially in endemic areas with poor sanitation.

## Consent

Written informed consent was obtained from the patient for publication of this case report and accompanying images. A copy of the written consent is available for review by the Editor-in-Chief of this journal on request.

The patient gave the official and ethical approved, informed consent for this publication and images.

## Ethical approval

Ethical approval is exempt/waived at our institution.

## Funding

Not applicable.

## Author contribution

Dr. Behzad Nemati Honar is the main author and he has designed this report. He is our mentor and specialized general surgeon.

Dr. Seyed Pedram Kouchak Hosseini is the senior resident of general surgery and he collected datas.

Dr. Alireza Haghbin Toutounchi is the intern of surgery and the writer of this article and coresponding author.

## Guarantor

Dr. Behzad Nematihonar accepts all responsibility of this article.

## Research registration number

Not applicable.

## Declaration of competing interest

All authors declare that they have no conflicts of interest.
